# Microglial extracellular vesicles’ proteome sheds light on the mechanisms underlying Alzheimer’s disease progression

**DOI:** 10.1002/alz.091884

**Published:** 2025-01-03

**Authors:** Martina Gabrielli, Elisa Tonoli, Elisabetta Battocchio, Clare Coveney, David Boocock, Ottavio Arancio, Elisabetta A M Verderio, Claudia Verderio

**Affiliations:** ^1^ CNR Institute of Neuroscience, Vedano al Lambro Italy; ^2^ Nottingham Trent University, Nottingham United Kingdom; ^3^ University of Milano‐Bicocca, Milan Italy; ^4^ Columbia University, New York, NY USA; ^5^ University of Bologna, Bologna Italy

## Abstract

**Background:**

We recently demonstrated that large extracellular vesicles (EVs) released by Aβ‐loaded microglia and carrying Aβ (Aβ‐EVs) propagate synaptic dysfunction in the mouse brain by moving at the axon surface (Gabrielli et al., Brain, 2022; Falcicchia et al., Brain Commun, 2023). Compared to ctrl‐EVs, not carrying Aβ, a higher number of Aβ‐EVs move along axons and travel longer distances through a yet undefined mechanism. Our previous work indicates that large EVs motion can be passively driven by neurons, via EV interaction with a neuronal receptor linked to the cytoskeleton, or may happen autonomously when EVs contain actin filaments and ATP in their lumen. To get insights into the mechanisms underlying higher extracellular Aβ‐EVs motion, we here analysed microglial EVs proteome.

**Method:**

Aβ‐EVs were obtained from primary microglia exposed to Aβ_42._ High‐resolution accurate‐mass spectrometry SWATH™‐MS followed by DIA‐NN quantitative analysis was employed to identify differentially expressed proteins in Aβ‐ vs. ctrl‐EVs, which may justify Aβ‐EVs empowered motility and describe the molecular mechanisms underlying EVs extracellular motion. Western blot and optical manipulation coupled to time‐lapse imaging were also employed to explore the involvement of additional candidate molecules, such as transglutaminase 2 (TG2).

**Result:**

A total of 148 proteins were upregulated in Aβ‐ compared to ctrl‐EVs, while 169 were downregulated. Panther GO analysis showed that terms enriched in large Aβ‐EVs include cytoskeletal protein binding (GO:0008092) and ATP hydrolysis activity (GO:0016887), suggesting that a higher fraction of Aβ‐EVs may undergo independent motion. STRING analysis revealed a high protein‐protein interaction (PPI) among Aβ‐EVs enriched molecules, some of which are differentially expressed also in EVs from AD/MCI patients or AD mouse models, validating the relevance of our findings.

**Conclusion:**

Our data suggest that Aβ‐EVs higher motility and capability to propagate synaptic dysfunction may be due to their augmented capacity to undergo active motion, and identify novel proteins potentially involved in EVs motion. Further analysis will be necessary to validate these findings in AD.

**Fundings**: 2018‐AARF‐588984, Horizon2020#874721PREMSTEM.

